# Characterizing carrier transport in nanostructured materials by force-resolved microprobing

**DOI:** 10.1038/s41598-020-71147-y

**Published:** 2020-08-25

**Authors:** Yen Nguyen, Hui-Ping Chang, Meng-Syun Hsieh, Ian Daniell Santos, Sheng-Ding Chen, Ya-Ping Hsieh, Mario Hofmann

**Affiliations:** 1 Graduate Institute of Applied Physics, National Taiwan University, Taipei, 10617 Taiwan; 2 Institute of Atomic and Molecular Science, Academia Sinica, Taipei, 10617 Taiwan; 3 Department of Materials Science and Engineering, National Cheng Kung University, Tainan, 70101 Taiwan; 4 Department of Physics, National Taiwan University, Taipei, 10617 Taiwan

**Keywords:** Materials science, Nanoscience and technology

## Abstract

The advent of novel nanostructured materials has enabled wearable and 3D electronics. Unfortunately, their characterization represents new challenges that are not encountered in conventional electronic materials, such as limited mechanical strength, complex morphology and variability of properties. We here demonstrate that force-resolved measurements can overcome these issues and open up routes for new applications. First, the contact resistance to 2D materials was found to be sensitively depending on the contact force and, by optimizing this parameter, reliable contacts could be repeatably formed without damage to the fragile material. Moreover, resistance of three-dimensional surfaces could be investigated with high accuracy in spatial position and signal through a force-feedback scheme. This force-feedback approach furthermore permitted large-scale statistical characterization of mobility and doping of 2D materials in a desktop-sized automatic probing system that fits into glove boxes and vacuum enclosures using easily available and low-cost components. Finally, force-sensitive measurements enable characterization of complex electronic properties with high lateral resolution. To illustrate this ability, the spatial variation of a surface’s electrochemical response was investigated by scanning a single electrolyte drop across the sample.

## Introduction

Recent years have seen a revolution in electronics concerning both the range of available materials and the capabilities of novel devices. Nanostructured materials, such as 2D materials, nanowires, organic polymers, and functional molecules have demonstrated unprecedented properties and abilities in carrier conduction, sensing, and information processing^1–7^. These advances have been employed to produce novel wearable, high performance, and large scale electronic devices supporting the vision of ubiquitous electronics.

However, the advances of nanostructured materials come at the cost of increased complexity compared to traditional semiconductor materials. First, the mechanical strength of nanostructures is limited, which complicates their handling^8–10^. Secondly, many of the unique properties are derived from complex and non-planar morphologies, such as nanowire solar cells, electronic textiles, and 3D printed electronic devices^11,12^. Finally, current synthesis approaches yield mixtures of properties, such as chirality, dimensions, and variable extrinsic interactions that complicate engineering design^13^.

To address these challenges and enhance the understanding of nanostructured materials, a comprehensive electrical characterization approach is required that permits investigation on large device numbers, retains the structure of nanomaterial, and is compatible with different measurement techniques. Unfortunately, to date, electrical measurements of nanostructures have followed conventional semiconductor probing approaches. Metallic probe tips have been employed to make electrical contact to nanostructures by identifying their point of contact by visual inspection^14,15^, electrical feedback^16–19^, shear force characterization^20^ and deflection^18,21^. While such techniques can reliably produce electrical contact, they are limited in their suitability for the envisioned application of nanostructure characterization. First, many deformable probe designs cannot easily be adapted to variable electrode spacing or electrode geometries and can only investigate microscopic regions. Moreover, electrical feedback systems are not compatible with high-resistance nanostructures. Finally, visual inspection is challenging for complex sample morphologies or transparent samples.

We here extend the capabilities of electrical probing towards adaptability to complex and dynamically changing morphologies, retaining the structural integrity, and permitting measurements on statistically relevant scales by a force-resolved approach. This advance is achieved by combining electrical probing and indentation techniques that analyze the force–displacement characteristics of a material. Indentation has demonstrated the ability to extract important information about the mechanical properties of complex nanostructured materials^22^ preserve fragile morphologies^23^, and conduct statistical evaluation^24^, which are attractive features for electrical probing.

A simple and extendable setup was designed that permits the reliable, scalable, and powerful investigation of nanostructured materials. Using a force-resolved probing approach, the interaction of contacts with nanostructured materials was optimized and a force regime was identified that enables the reliable and repeatable measurement without incurring damage on 2D materials and nanostructured films. Moreover, force-resolution enables a facile large-scale-mapping process to investigate materials properties on morphologically complex and compositionally varying materials and permits statistical analysis of 2D material properties. Finally, force-resolved approaches open up routes for advanced carrier transport characterization as illustrated through the example of spatially resolved electrochemical mapping measurements that exhibit high lateral resolution. Our results open up new routes towards characterizing nanostructured materials and applying them in future electronic devices.

## Results and discussion

### The relationship between contact resistance and loading force

The contact between microscopic probe tips and electrodes has been considered from a tribological perspective, i.e. asperities and roughness prevents intimate contact and pressure has to be applied to homogenize these contact points^25^. Consequently, previous reports observed a continuous and inverse exponential relationship between normal force and contact resistance^26,27^. To test this assumption for nanostructured films, we investigated the resistance of a planar 50 nm Au film on top of single-layer graphene—a two-dimensional carbon allotrope—at different loading conditions.

**Figure 1:**
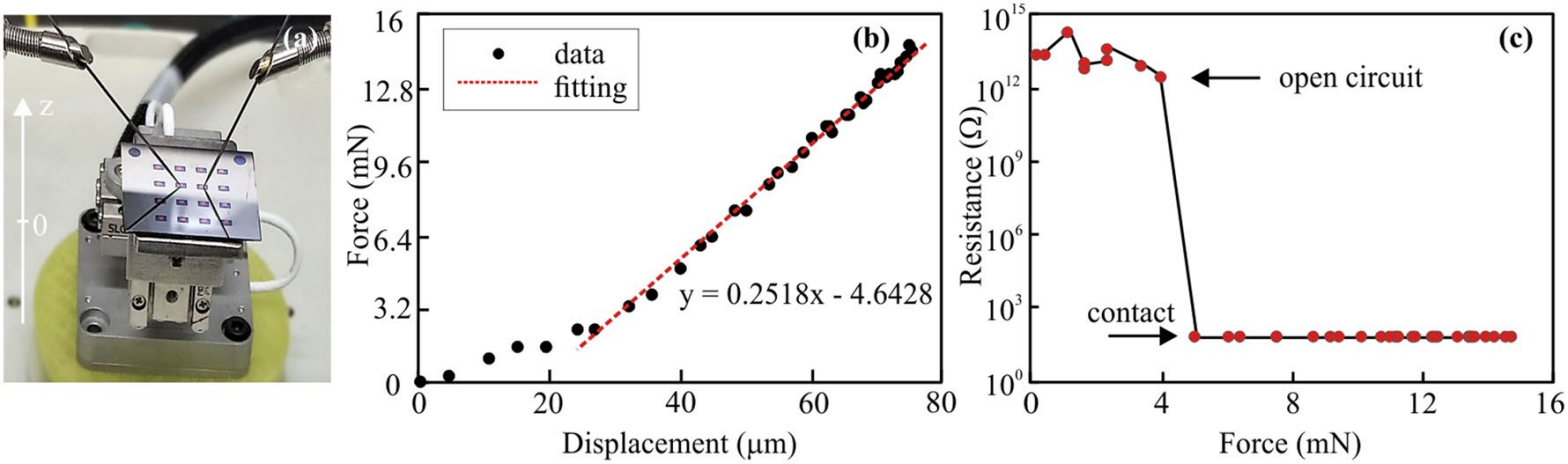
Clarification of force-sensitivity measurement (**a**) Photo of measurement setup, (**b**) Force–displacement plot, dots are measured data, black line is fitting result, (**c**) Relationship between resistance and force before and after contact have been made.

For this purpose, the sample and a piezoelectric actuator were attached to a load cell and the sample was raised towards stationary microprobe tips (tungsten, 0.51 mm diameter, 38 mm length) (Fig. 1a). (A detailed description of the system can be found in the Supporting Information). The force–displacement curve shows an initially lower slope due to an angular misalignment of the sample and the probes of 10 mrad and the large-displacement region can be well fitted by considering a spring constant of 251.8 N/m (Fig. 1b). This value for the spring constant agrees well with simple Euler–Bernoulli beam calculation for the employed tips (see Supporting Information for more details). Subsequently, two probes were positioned on top of the Au film and the film resistance between the two probes was measured at varying displacements, resulting in different applied loads. We observe a sharp transition between open circuit and low resistance regimes at a force of approximately 4 mN (Fig. 1c). This transition indicates that contact is made abruptly, despite the macroscopic size of the contacting tips. More importantly, we observe no improvement in contact resistance even if the contact force is tripled. Moreover, upon increasing the force tenfold (Figure S1), the resistance increased again to 2.2 × 10^11^ Ω, which is clear evidence of the device failure under high loads. This is an important observation, because it emphasizes the merit of force-sensitivity since the displacement between open-circuit and contact is only 5 µm, which is close to the resolution of inspection microscopes employed in conventional probe placement schemes.

### Force resolved measurement with Force feedback loop integration

**Figure 2:**
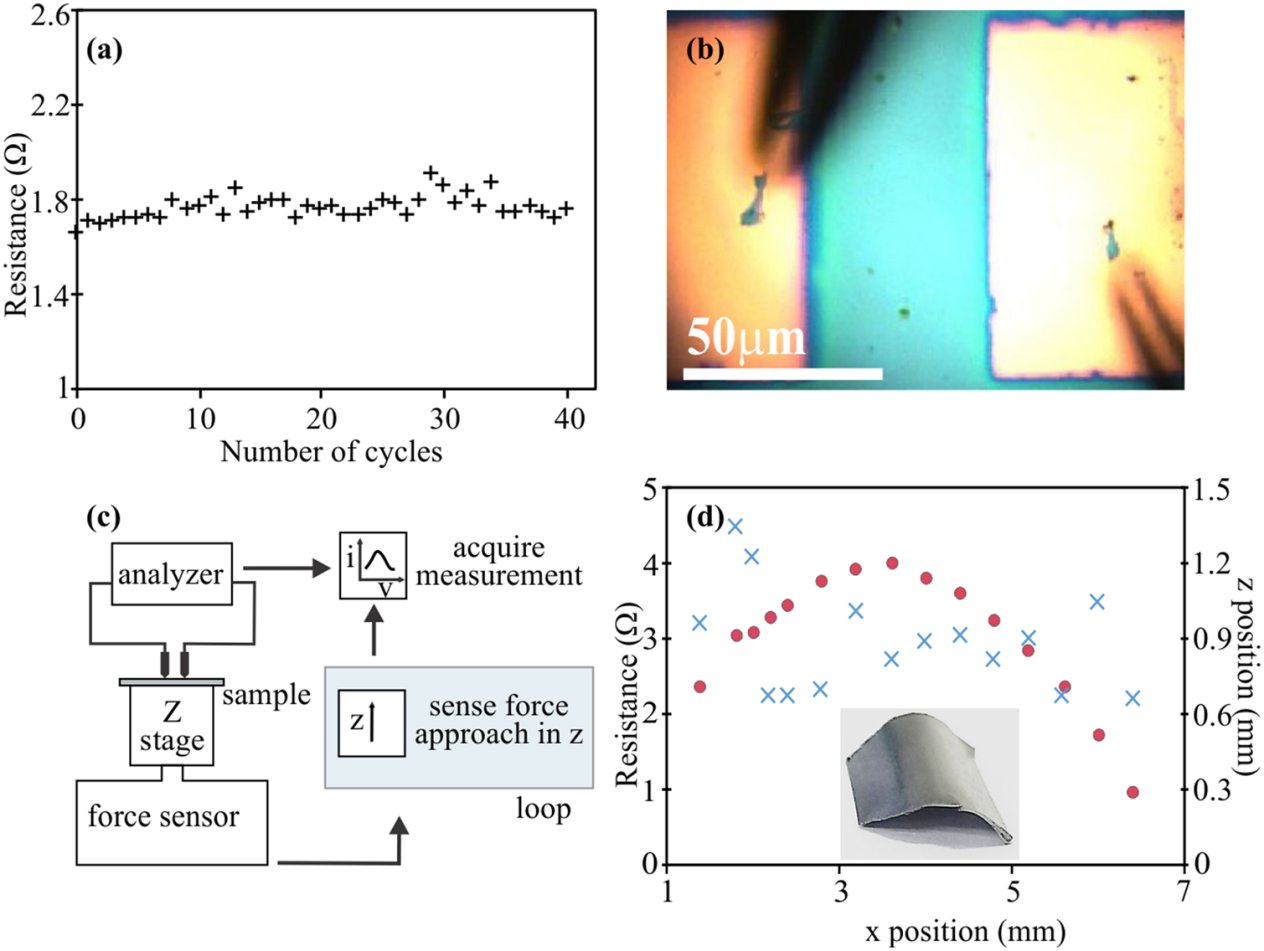
(**a**) Resistance after multiple load-unload cycles, (**b**) Optical image of damaged electrodes after 50mN of loading force, (**c**) Schematic diagram of force-sensitive system integrated with force-feedback loop, (**d**) Operation on a convex surface, red dots delineate the surface of sample in z direction and corresponded resistance in blue cross markers.

To demonstrate the importance of force-sensitivity, we conduct cyclical loading measurements for two loading conditions (Fig. 2a). We observe that forces close to the contact point (i.e. 5 mN) can be employed to reliably contact the graphene/Au film for more than 40 times whereas a higher load of 50 mN results in failure after the first cycle as confirmed by micrographs of the damaged electrodes (Fig. 2b). This observation suggests the usage of small force to minimize the destruction of contact pads is instrumental in conducting reliable measurements.

Based on this understanding, we integrate the force-sensing probe system with a feedback loop, that stops the approach between sample and tips when the proper load force is achieved (Fig. 2c). Due to the high resolution of the employed actuator, displacement accuracies of 100 nm could be achieved, permitting the reliable measurement even on complex 3D morphologies. We demonstrate the feasibility of the probing by measuring conductivity on a convex metal surface. Lateral offset resulted in largely varying height values at which reliable contact was achieved and consequently, the measured displacement traces the shape of the surface (Fig. 2d). Despite the complex morphology, resistance variations are 23% and a systematic increase in error is only observed in regions of the sample that are not accessible to both probes. This result demonstrates the potential of applying force-sensitive probing to simultaneously characterize the morphology and electrical properties of complex nanostructure devices.

### Automated mapping of 2D materials electrical properties

**Figure 3:**
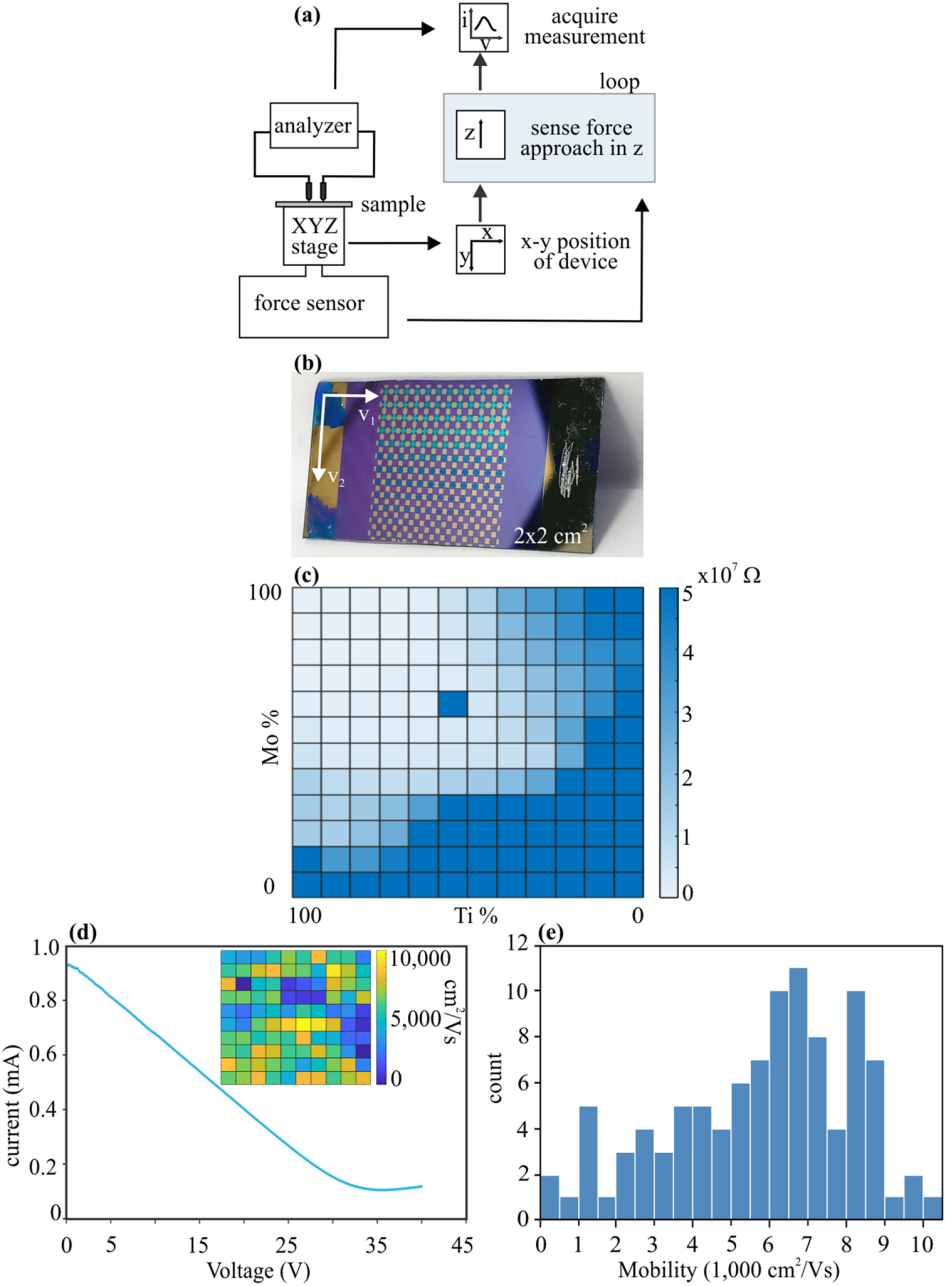
Large scale mapping of materials electrical characterization (**a**) Schematic diagram of operation, (**b**) Photograph of 144 devices fabricated on two-dimensional CVD Ti-MoS2 film, (**c**) Statistical conductivity over 144 devices as function materials composition, (**d**) Electrical transport characterization for graphene FET, inset is mobility mapping for 100 devices, (**e**) Mobility distribution histogram.

The presented feedback system combining with an x–y positioning system enables the realization of facile automatic probing systems that can be used for the statistical evaluation of a material’s electronic properties (Fig. 3a). Compared to conventional microprobing systems, which are a mainstay in commercial semiconductor fabrication, the use of our approach has several advantages. First, the use of a force-feedback system, instead of conventional optical focus sensors, reduces the systems complexity and simplifies operation. Moreover, the previously demonstrated ability to finely adjust the loading force, results in a decreased chance of damage compared to traditional spring-loaded microprobes.

To demonstrate the utility of our method, we investigate large arrays of 2D materials devices in a reliable and straightforward manner. Figure 3b shows a photograph of a sample that contains 144 devices produced from chemical vapor deposition (CVD)-grown MoS_2_. MoS_2_ is a well-known two-dimensional transition metal dichalcogenide with substantial potential for future electronic devices^28,29^. Co-deposition of Mo and Ti resulted in spatial variation of materials resistance as described in the methods section. Probing of large arrays of devices can be achieved by defining a lattice through linear combination of two translation vectors v_1_, v_2_ as shown in Fig. 3b. The two vectors are obtained in a convenient fashion by defining three positions on the array corners, namely top left, top right and bottom right and inputting the number of devices between two selected points. Figure 3c is the heatmap of resistance over four square centimeters demonstrating that 100% of devices were successfully probed despite their large resistance. We observe a smooth change in conductivity that correlated with the variation of the materials composition and highlights the potential of our approach to characterizing large numbers of 2D materials devices.

We furthermore demonstrate lateral mapping of more complex electronic properties, by conducting transconductance measurements of 100 graphene field effect transistors. For this purpose, two terminals were deposited onto the graphene film and a global back gate was employed.

Drain current measurements during bottom gate voltage sweeps V_g_ from 0 to 40 V were obtained at V_ds_ = 1 V. Figure 3d shows the ambipolar characteristics of the graphene devices, in agreement with previous reports. The p-type transfer characteristics are observed, which originates from the environmental doping of graphene by oxygen and water molecules. The mobility μ_k_ can be extracted by fitting the data to the following equation:

$${\upsigma }_{\mathrm{ds}}{\mathrm{V}}_{\mathrm{g}}={({\left({\mathrm{C}}_{\mathrm{g}}{\upmu }_{\mathrm{k}}\left|{\mathrm{V}}_{\mathrm{g}}-{\mathrm{V}}_{\mathrm{D}}\right|+{\upsigma }_{\mathrm{bg}}\right)}^{-1}+{\uprho }_{\mathrm{r}})}^{-1}$$

where σ_ds_ is sheet conductivity I_ds_ L/(V_ds_ W), L is length of channel, W is width of channel, C_g_ is gate capacitance (for 300 nm SiO_2_, C_g_ = 1.15 × 10^–8^ Fcm^−2^), σ_bg_ is the background conductivity, ρ_r_ is the residual resistivity. V_D_ is the charge neutrality point that presents the amount of external doping in graphene. Notably, the mobility map exhibits the diversity as in inset, which can be ascribed to inhomogeneous doping throughout the graphene layer. The histogram in Fig. 3e describes more detailed the distribution of mobilities.

### Electrochemical characterization

**Figure 4:**
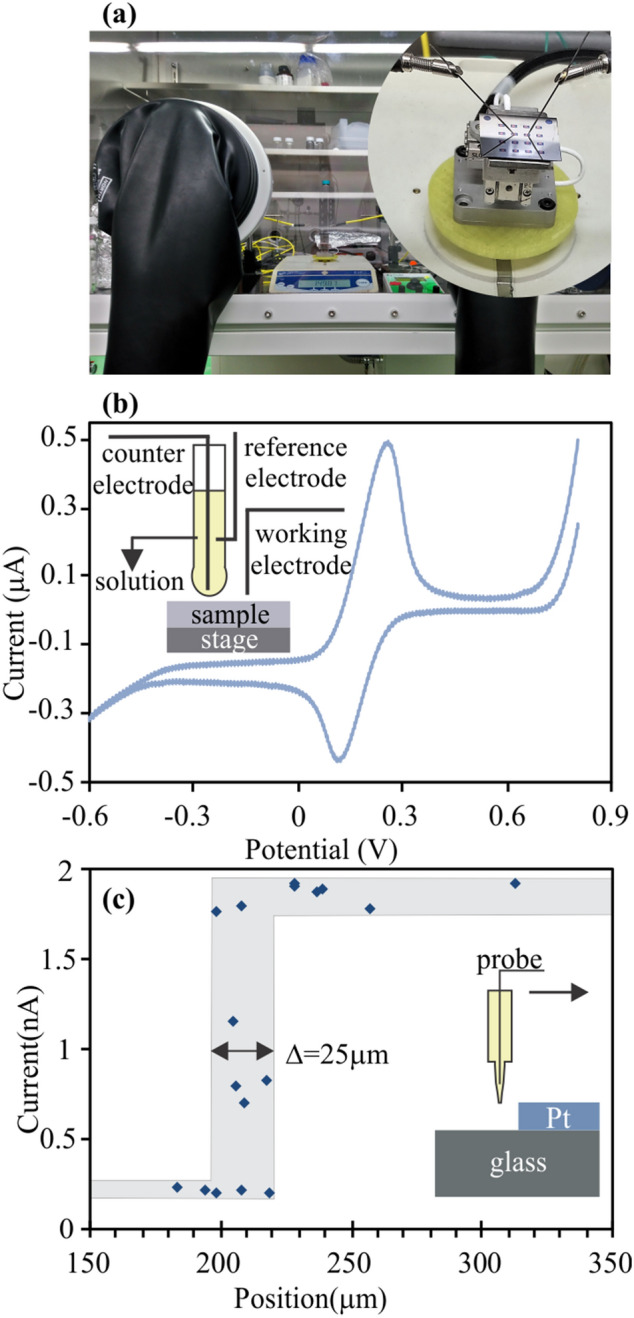
(**a**) The prober is well-fitted in the glove box, the inset is contacting process of probe tips and the sample rested on the positioning stage, (**b**) Cyclic Voltammetry measurement in a droplet, inset is the setup with working electrode, reference electrode, counter electrode and electrolyte, (**c**) Experiment setup to define the resolution of pipette, arrow indicates movement direction of probe.

Our force-sensitive probing approach permits the proliferation of reliable force-sensitive measurements on complex morphologies over large scale to researchers worldwide. Since no complex optical alignment system is needed, our system can be easily realized with three off-the-shelf components as described in the supplementary material for a fraction of the cost of a traditional probing system. The complete setup exhibits an extremely small footprint with dimensions below 200 × 300 × 300 mm^3^, permitting integration into environmental chambers and glove boxes as shown in Fig. 4a. Moreover, due to a simple interface, users can modify the setup according to different research directions.

Finally, we demonstrate the ability to extend force-sensitive and laterally resolved measurements beyond conventional carrier measurements. For this purpose, spatially resolved electrochemical characterization is conducted using a single microscopic electrolyte droplet. Both counter and reference electrode are inserted into a droplet that is suspended on a micropipette (inset Fig. 4b), thus forming a complete electrochemical cell when brought in contact with a substrate. The operation of the cell was demonstrated through a cyclic voltammogram of a single electron reduction and oxidation within a potential window from − 0.6 V to 0.8 V. The obtained voltammogram is in agreement with previous reports and shows the potential of droplet-based electrochemistry^30,31^.

As an extension of previous reports on droplet-enabled electrochemistry^32,33^, we employ force-sensitivity to repeatedly bring the droplet in contact with the substrate at different positions (more information on the setup and measurement processes are provided in the supplementary material). This automated positioning enables the spatially resolved measurement of electrochemical properties as demonstrated by measuring the peak CV current as a function of displacement relative to the edge of a platinum thin film (Fig. 4c). The probe moves from the glass to Pt side, leading to very small current at the beginning due to non-conductive glass. The current, however, scatters from the position of 195 µm to 220 µm and then keeps stable at a high value. The scattering area Δ indicates that the probe crosses the edge between non-conductive and conductive area and the width of the transition region (Δ = 25 µm) agrees well with the dimension of the micropipette opening. Thus, minimization of pipette opening^34^ could further enhance the resolution and permit the selective investigation of the electrochemical response of nanometer-sized structures.

## Methods

### Graphene FET

Graphene was synthesized by CVD method. A large copper foil of 2 × 7 cm^2^ (99.8%, Alfa-Aesar) was electropolished following by annealing under a flow of 200 sccm H_2_ at 1,000 °C for 30 min. Next, 10 sccm CH_4_ was introduced at the pressure of 9 Torr to initiate graphene growth; the growth duration is 6 h. The grown sample was then cooled down to room temperature in a 10 sccm H_2_.

In the next step, graphene was transferred into doped Silicon wafer with 300 nm SiO_2_ insulator through wet transfer method. Briefly, we coated a thin layer of PMMA on graphene and dried them on a hotplate at 60 °C for 30 min. Copper was etched by APS etchant, leaving PMMA/graphene film afloat. The film was cleaned multiple times by DI water before it was transferred into a prepared substrate. After drying process, we removed PMMA by using acetone and cleaned the sample again with isopropyl alcohol. The source and drain electrodes of 50 nm Au were created by thermal evaporation through G-200 TEM mask.

### Combinatorial CVD Ti-MoS_2_ devices

We also prepared MoS_2_ thin film with Ti doping via CVD method. Ti and Mo were deposited on 300 nm SiO_2_/silicon wafer by e-beam evaporation through a gradient mask. The content of Mo and Ti was much richer at the top left and gradually reduced to very little at the bottom right of sample. The as prepared substrate was put into 1-inch quartz tube furnace using sulfur powder as a precursor. Initially, the quartz tube was purged several times with Ar gas (flow rate of 200 sccm). During the growth run, a mixture of 100 sccm Ar and 40 sccm H_2_ was introduced into the tube and the furnace temperature increased to 950 °C. The growth process was at atmospheric pressure for 40 min. The sample was cooled down naturally. Ti/Au (10 nm/50 nm) electrodes were deposited by e-beam evaporation through a shadow mask. All the electrical investigation of graphene and MoS_2_ was carried out by a semiconductor analyzer (HP-4156B).

### Preparation of cyclic voltammetry

The substrate was prepared by deposition of 50 nm Pt on a glass via e-beam evaporator. The counter electrode is Pt, reference electrode is Ag/AgCl. The solution for CV experiments consists of 10 mM K_3_Fe(CN)_6_ and 0.1 M KCl. The electrochemical characterization was conducted by a CHI-660 potentiostat.

## Conclusion

In conclusion, we have introduced a novel approach to characterize electrical properties of nanostructured and mechanically fragile materials with complex morphologies. Force-resolved electrical microprobing was shown to produce sensitive, highly accurate and stable contacts for statistical evaluation of large arrays of graphene and MoS_2_ devices to extract statistical information on doping and mobility. Moreover, the described force-feedback system can be employed for characterization of diverse properties, and spatially resolved electrochemical microscopy of a single droplet were demonstrated. Finally, our system can be easily reproduced by researchers and extended to a wide variety of applications and environments, such as glove boxes and vacuum enclosures.

## Supplementary information

Supplementary Information
