# Supplementary material for: Characterizing carrier transport in nanostructured materials by force-resolved microprobing
Source: Sci Rep. 2020 Aug 25;10:14177. doi: 10.1038/s41598-020-71147-y (PMC7447755; doi:10.1038/s41598-020-71147-y)
Supplement: Supplementary file 1 — Supplementary Information [file 41598_2020_71147_MOESM1_ESM.docx]

Supporting Information

**Characterizing carrier transport in nanostructured materials by force-resolved microprobing**

**Yen Nguyen^1^, Hui-Ping Chang^2^, Meng-Syun Hsieh^3^, Ian Daniell Santos^4^, Sheng-Ding Chen^4^, Ya-Ping Hsieh^2^, Mario Hofmann^4*^**

^1^Graduate Institute of Applied Physics, National Taiwan University, Taipei 10617, Taiwan

^2^Institute of Atomic and Molecular Science, Academia Sinica, Taipei 10617, Taiwan

^3^Department of Materials Science and Engineering, National Cheng Kung University, Tainan, 70101, Taiwan

^4^Department of Physics, National Taiwan University, Taipei 10617, Taiwan

***Corresponding author:**

**Prof. Mario Hofmann**

Email: mario@phys.ntu.edu.tw

**Components of Force-resolved measurement system**

The force sensor is based on a laboratory scale from Denver Inc. SI-234 with a weight range of 230g and a resolution of 0.1 mg. The scale connects with computer via RS-232 adapter to control weight querying during the contact process. A SmartAct piezo-based positioning stage is mounted on the top of the laboratory scale allowing motions of the sample in three dimensions. This positioner achieves travel within a range of 18 mm with a step size of 100 nm and a repeatable positioning of 500 nm. The communication between PC and positioner was facilitated through a USB interface. Manual manipulators (Keithlink) with probe arms and tungsten tips were magnetically attached on a frame that was separated from the scale assembly. They allow fine controllable motion of 12mm in three dimensions. The electrical measurements are carried out by a semiconductor analyzer (HP-4156B)

**Different loading comparison**

Under safe loading of 5mN, the resistance has measured to be 1.7Ω as in Figure S1(a)(b). For high force probing, the optical image shows damage of Au electrode, as a consequence the contacting has been lose the resistance increases to 2.2x10^11^Ω


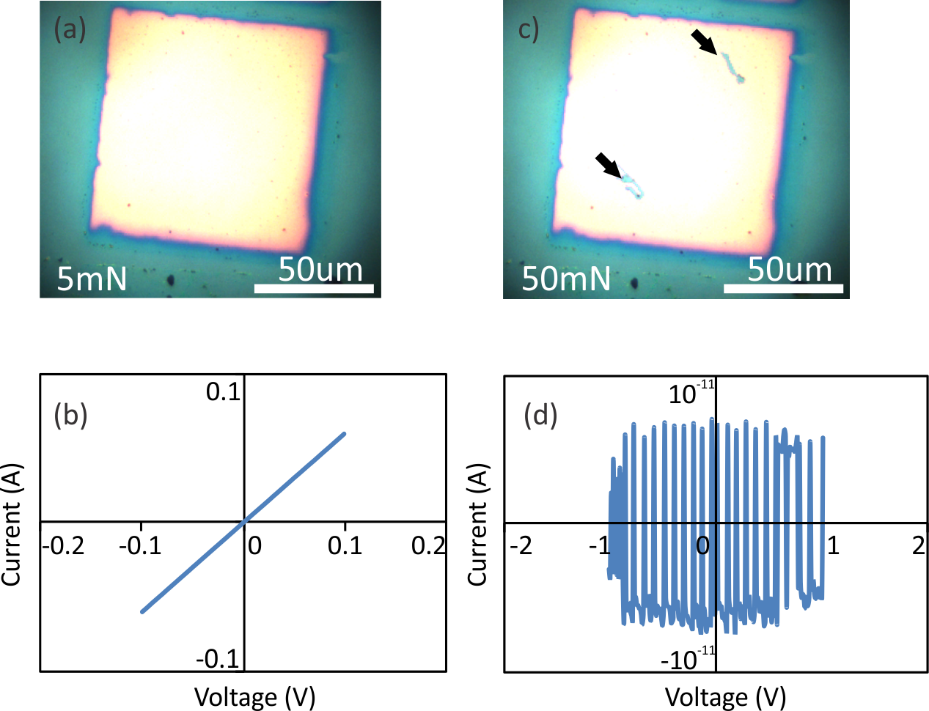


**Figure S1:** (**a**)(**c**) Optical micrograph of Au electrodes after different loading force, (**b**)(**c**) Corresponding IV has been measured demonstrating the damage due to high load

**Electrochemical setup**

Both reference and counter electrode are immersed in the electrolyte inside a syringe. The syringe tip is attached to a micropipette through a plastic adapter. The amount of solution is well controlled by syringe pump to account for evaporating electrolyte during the whole measurement.


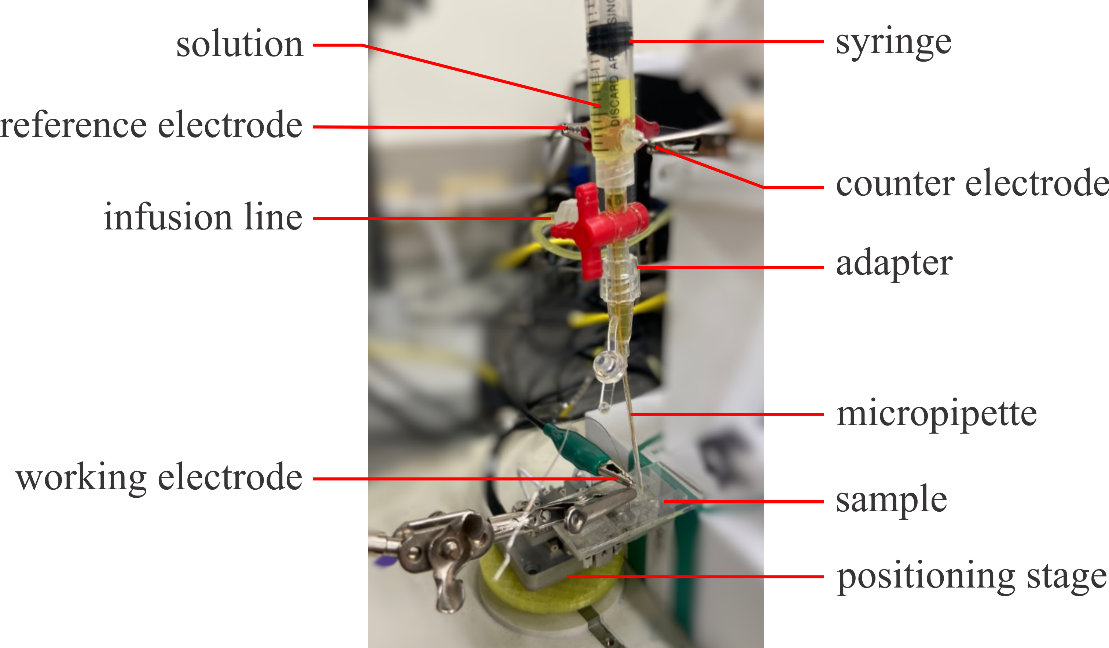


**Figure S2:** Setup for automated electrochemical measurement

**Force-displacement measurement for a droplet probe**

Similar to the case of using tungsten tip, we also have performed force sensitivity to find contact condition for the droplet probe. At large displacements the slope is comparable to the experiments with metallic probes. However, for small displacements the slope is significantly smaller, indicating the easy displacement of the liquid droplet at the probe tip


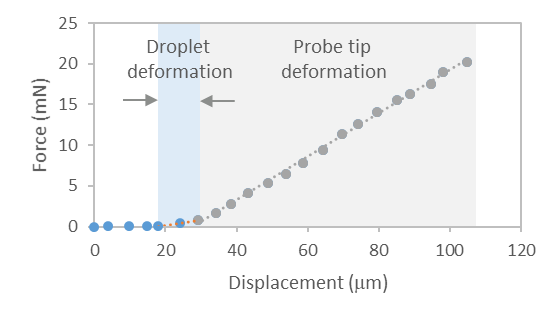


**Figure S3:** Force-displacement curvature for a droplet probe

**Calculate stiffness of probe tip**

Using Euler–Bernoulli beam model to calculate stiffness of tungsten tip

$$k=\frac{3E\pi R^{4}}{2L^{3}}=149.17(N/m)$$

where E=4.11x10^11^(N/m^2^) is the Young’s modulus of the tungsten probe, R is its radius and L is its length.
